# Obstetric operating room staffing and operating efficiency using queueing theory

**DOI:** 10.1186/s12913-023-10143-0

**Published:** 2023-10-25

**Authors:** Grace Lim, Annamarie J. Lim, Beth Quinn, Brendan Carvalho, Mark Zakowski, Grant C. Lynde

**Affiliations:** 1 Department of Anesthesiology & Perioperative Medicine, University of Pittsburgh, 300 Halket Street #3510, Pittsburgh, PA 15215 USA; 2 Department of Obstetrics & Gynecology, UPMC Magee-Womens Hospital, University of Pittsburgh, Pittsburgh, PA USA; 3 Schumacher Clinical Partners (SCP) Health, Traverse City, MI USA; 4 Department of Anesthesiology, Perioperative and Pain Medicine, Stanford University, Stanford, CA USA; 5 Cedars-Sinai Medical Center, Los Angeles, CA USA; 6 Hospital Corporation of America (HCA) Healthcare, Nashville, TN USA

**Keywords:** Staffing, Obstetric, Anesthesia, Efficiency, Queueing, Operating room

## Abstract

**Introduction:**

Strategies to achieve efficiency in non-operating room locations have been described, but emergencies and competing priorities in a birth unit can make setting optimal staffing and operation benchmarks challenging. This study used Queuing Theory Analysis (QTA) to identify optimal birth center operating room (OR) and staffing resources using real-world data.

**Methods:**

Data from a Level 4 Maternity Center (9,626 births/year, cesarean delivery (CD) rate 32%) were abstracted for all labor and delivery operating room activity from July 2019—June 2020. QTA has two variables: Mean Arrival Rate, λ and Mean Service Rate µ. QTA formulas computed probabilities: P_0_ = 1-(λ/ µ) and P_n_ = P_0_ (λ/µ)^n^ where *n* = number of patients. *P*_*0…n*_ is the probability there are zero patients in the queue at a given time. Multiphase multichannel analysis was used to gain insights on optimal staff and space utilization assuming a priori safety parameters (i.e., 30 min decision to incision in unscheduled CD; ≤ 5 min for emergent CD; no greater than 8 h for *nil per os* time). To achieve these safety targets, a < 0.5% probability that a patient would need to wait was assumed.

**Results:**

There were 4,017 total activities in the operating room and 3,092 CD in the study period. Arrival rate λ was 0.45 (patients per hour) at peak hours 07:00–19:00 while λ was 0.34 over all 24 h. The service rate per OR team (µ) was 0.87 (patients per hour) regardless of peak or overall hours. The number of server teams (s) dedicated to OR activity was varied between two and five. Over 24 h, the probability of no patients in the system was P_0_ = 0.61, while the probability of 1 patient in the system was P_1_ = 0.23, and the probability of 2 or more patients in the system was P_≥2_ = 0.05 (P_3_ = 0.006). However, between peak hours 07:00–19:00, λ was 0.45, µ was 0.87, s was 3, P_0_ was 0.48; P_1_ was 0.25; and P_≥2_ was 0.07 (P_3_ = 0.01, P_4_ = 0.002, P_5_ = 0.0003).

**Conclusion:**

QTA is a useful tool to inform birth center OR efficiency while upholding assumed safety standards and factoring peaks and troughs of daily activity. Our findings suggest QTA is feasible to guide staffing for maternity centers of all volumes through varying model parameters. QTA can inform individual hospital-level decisions in setting staffing and space requirements to achieve safe and efficient maternity perioperative care.

## Key points

*Question* Can Queueing Theory Analysis (QTA) be used to assess appropriate obstetric operating room staffing models and efficiency at maternity hospitals?

*Findings* QTA can be used to inform birth center operating room staffing models and to gauge efficiency.

*Meaning* QTA methods are one way to provide data-driven information to guide individual hospital-level decisions in setting obstetric operating room staffing requirements.

## Introduction

Hospital-based maternity centers often face operational challenges in balancing safety and efficiency. Surge response capability (SRC), the ability to accommodate a surge in clinical emergencies or periods of high volume, is a principle applied in mass casualty incidents [1] that can also translate to obstetric operations. Maintaining efficient staffing and resources while always remaining prepared for obstetric emergencies makes it essential to strike the optimal balance in SRC that is not so low that it poses a threat to patient safety and not so high as to create work inefficiencies.

Updated recommendations by the American College of Obstetricians and Gynecologists (ACOG) highlight these efficiency challenges through Levels of Maternal Care [2]. These levels specify different services, capabilities, and health care providers, such that Level 1 (Basic Care) cares for low- to moderate-risk pregnancies with emergency providers “available at all times” while Level 4 (Regional Perinatal Health Care Center) calls for on-site availability or presence of nursing, maternal–fetal medicine specialists, specialty surgeons, and obstetric anesthesiologists. Society for Obstetric Anesthesia and Perinatology (SOAP) Centers of Excellence also specify obstetric anesthesia staffing as criteria. Despite these highly visible societal recommendations, there is little available data to objectively guide appropriate staffing for a given maternity center needs. Although operating efficiency in non-operating room hospital locations has been described [3], emergencies and competing dynamic priorities in a birth unit can make it challenging to set optimal staffing and operation benchmarks. To our knowledge, there have been no studies on efficiency models specific to maternity centers that can guide clinicians, administrators, and payers in decision-making for appropriate obstetric staffing needs.

Queueing theory analysis (QTA) is a mathematical concept used in the study of congestion and delays from waiting in lines. It can help healthcare stakeholders make informed decisions on creating safe, efficient, and cost-effective workflow systems, such as mass casualty events [4, 5]. QTA has also been used in emergency department models to develop flow models that optimize wait times [6]. QTA includes probabilities of arrival in the “queue” for service – in this case, obstetric patients requiring care in a birth center operating room (OR) – then waiting in the queue, then being served – care in the OR. QTA allows assessment and observation of several metrics, including average wait time, expected number in queue, expected number receiving care, and the probability of the system being in several states (e.g., multiple rooms running and duration of overlapping time intervals). QTA insights require medical ground-level context because analyses explore trade-offs between the cost of hiring additional teams to provide care, versus the cost-of-service delays. The goal of QTA within the healthcare setting is to balance service in “surge,” or mass casualty incidents, and ordinary “non-surge” times of routine clinical care. QTA can thus be used as a tool to make decisions about SRC resources vis-à-vis surge capacity.

**Fig. 1:**
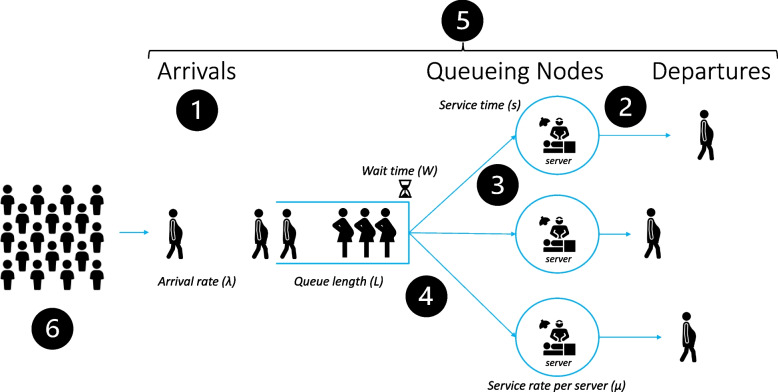
Conceptual diagram of queueing theory nodes and characteristics. Patients arrive at rate = *λ*, then await service for time = *w*, over queue length = *L*. Server teams are specified in analysis, with service time (*s*) defined as time required for treatment provided at service rate per server team = *µ*. Service nodes are varied in analysis to identify system performance for wait times and utilization. After service, patients then depart the system. Six distinct parameters are shown: 1) the arrival process; 2) the service and departure process; 3) the number of servers available; 4) the queueing discipline (in obstetric operations, the discipline is priority queue); 5) the queue/system capacity; 6) and the size of the client population

QTA involves persons arriving in a queue, waiting in the queue, receiving service, then departing the system (Fig. 1). QTA has two primary variables of interest: Mean Arrival Rate, calculated as patients per hour λ = number of patients per year divided by the number of hours; and Mean Service Rate µ = average length of cases in hours per patient / hour [7]. Both λ and µ can be calculated using historical or observed data. We abstracted this data from our medical records as described above. The arrival rate was defined using anesthesia start times; service rates were defined using OR times (anesthesia start and stop times). QTA formulas computed probabilities: P_0_ = 1-(λ/ µ) and P_n_ = P_0_ (λ/µ)^n^ where n = number of patients. *P*_*0…n*_ is the probability there are zero (P_0_) or one (P_1_) patients in the OR queue at a given time, and the probability that ≥ 2 patients require ORs simultaneously (P_2…n_). All probabilities add up to 1 (P_0_ + P_1_ + P_2_…P_n_ = 1). Therefore,

$$\begin{array}{c}\mathrm{P}\ge 2 = 1-\left({P}_{0}+{P}_{1}\right)\mathrm{ and}\\ \mathrm{P}\ge 2 = 1-((1-\uplambda /\upmu ) + [(1-\uplambda /\upmu )(\uplambda /\upmu )])\end{array}$$

This study aimed to explore the use of QTA to identify optimal birth center staffing and OR resources, using real-world data from a high-volume Level 4 maternity center.

## Methods

This computational study using retrospectively collected data was approved by the University of Pittsburgh Institutional Review Board (STUDY19120054). The requirement for informed consent was waived by the IRB. Data from this urban, high-volume Level 4 Maternity Center were abstracted for all OR activity from July 2019—June 2020. These OR activities included but were not limited to cesarean delivery (CD), tubal ligation, double set-up for vaginal twin delivery, dilation and curettage, external cephalic version, cerclage, fetal procedures, post-delivery procedures associated with bleeding such as laceration repairs, peri-delivery cystoscopies, exams under anesthesia with or without uterine balloon tamponade procedures, and procedures for retained placenta. Other information abstracted from the medical record included age, American Society of Anesthesiologists Physical Status (ASA PS), emergency designation by ASA PS, race, and obesity. Operational data were collected from medical records, including room locations, anesthesia start times, room start times, room end times, total room times, time of day, overlap times, and overlap frequencies.

We consider the application of principles of QTA to obstetric operations for this study. Utilization ratio (ρ) is defined as a ratio of demand for service to capacity as it changes throughout a workday. It is calculated as ρ = λ/µ and gives an understanding of the demand for resources. When ρ ≥ 1, a backlog develops and worsens. In the context of obstetric operations, an ideal ρ is not universally defined, but should ideally be low to allow minimal wait times and access to operative interventions to minimize risk for maternal or fetal harm. Setting a lower ρ also enables high variability in arrival rates and service times. Other parameters defined in our QTA included: the average number of people in the system (Ls), the average length of the queue or the average number of people in a line waiting for service (Lq), the average time for a patient in the system or waiting time plus service time (Ws), average time spent in the queue (Wq), the probability that the time spent in the queue is zero (Wq(0)), number of patients in the system and number of operating rooms in use in the system (n), and the probability that n patients require simultaneous OR service (P_n_). Although an ideal Wq(0) and P_n_ are not defined for obstetric operations, we defined a P_n_ < 1% and a Wq(0) no lower than 99% as acceptable parameters for clinical operations to optimize maternal–fetal safety and minimize the risk associated with delays.

In this study, we used the singular term “server team” to encompass all individuals required to staff one OR. This team consists of an anesthesia provider, circulator nurse, a perinatal nurse, a surgical technician, and an obstetrician with or without an assistant. At our institution, the anesthesia team in the birth center ORs follows a medical direction model consisting of an attending anesthesiologist plus a qualified hands-on provider (QHOP), i.e., resident, fellow, or nurse anesthetist. The anesthesia team is dedicated to the obstetric suite. The anesthesiologist oversees a maximum of 2 rooms when 1 or 2 are staffed with a resident; and, a maximum of 4 rooms if all rooms are staffed with nurse anesthetists. The model was run for the number of ORs in addition to the number of server teams. This model was not designed to speak to the availability of individual server components (e.g., availability of circulator, technician, nurse, etc.) in the ability to mount a surge response. Room turnover procedures, including cleaning and room preparations, are an average of 30 min at our institution but were not explicitly included in the model.

### Model assumptions

Safety parameters were defined by 30 min decision-to-incision in unscheduled non-emergent CD; ≤ 5 min for emergent CD; no greater than 8 h for *nil per os* time (for elective or non-emergent cases). The model assumed the following. To achieve these safety targets, a < 0.5% probability that a patient would need to wait was assumed. We assumed P_n_ < 1% and a Wq(0) no lower than 99%. These assumptions were made based on expert input from investigators (GL, GL) given the lack of established parameters for these targets specifically for obstetrics. In obstetric operations, the queueing discipline is priority queue (i.e., urgent deliveries requiring immediate service for maternal–fetal safety), and the size of the client population is assumed to be not amenable to adjustment or optimization (i.e., number of pregnant patients due for hospital service at any time). QTA parameters in obstetric operations that may be amenable for system optimization included the arrival process, the service and departure process, the number of servers available, and the queuing system capacity. Although other centers reported reductions in obstetric care utilization associated with the COVID-19 pandemic, that phenomenon was not observed in our cohort during this study period.

### Statistical analysis

Summary statistics for demographic information included mean and standard deviation for continuous variables following a standard normal distribution and frequencies with percentages for ordinal data. Multiphase multichannel analysis was used to gain insights on optimal staff and space utilization, assuming a priori assumptions and safety parameters. Target utilization ρ was set at > 0.10. We set ρ to above 0.10 to buffer against fluctuations in demand, allowing the system to temporarily handle spikes in traffic and variations in arrival rates that are inherent in obstetric operations, without overwhelming the system while preserving service quality and short wait times during busy periods. Poisson distribution of total operating room time was examined using histograms. Percentage of ORs overlapping were grouped by time of day and examined by histograms. Time intervals of overlaps were grouped (up to 15-min overlap, up to a 30-min overlap, and so on, until greater than 60-min overlap). Percentages of these overlaps by time interval group were examined by histograms. The number of server teams (s) dedicated to OR activity was varied between 2 to 5 between peak hours to assess the impact on all other model parameters. All analyses were performed using XLSTAT (Microsoft Inc., USA) and Stata SE 15.1 (StataCorp Copyright 1985, College Station, TX).

## Results

**Fig. 2:**
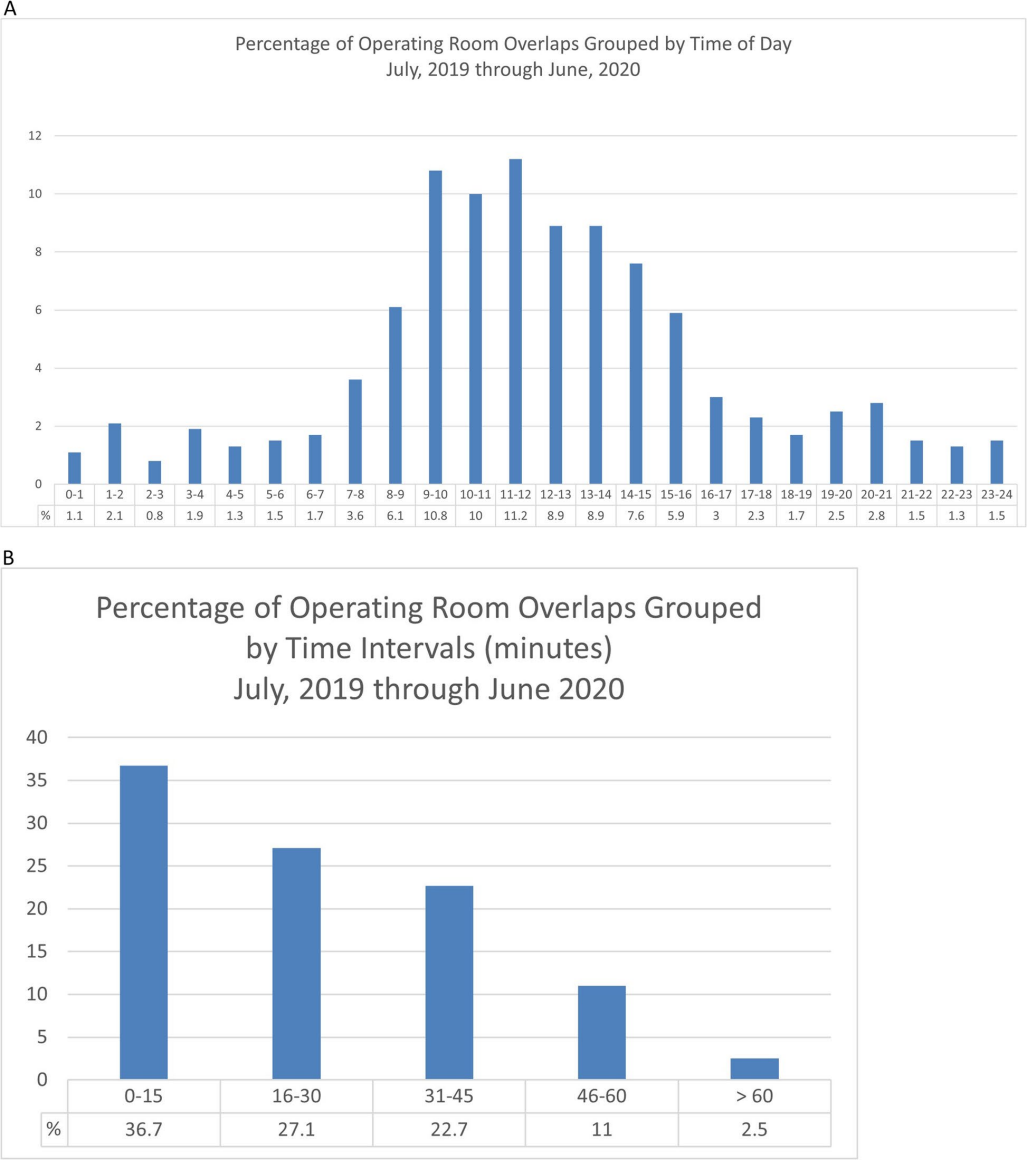
Characteristics of obstetric operating room activities by time of day and duration of overlaps. **A** Frequency distribution of 2 or more simultaneous operating room activities by time of day. **B** The same data depicted as frequency distribution of overlapping operating room activities by duration of overlap in minutes

**Fig. 3:**
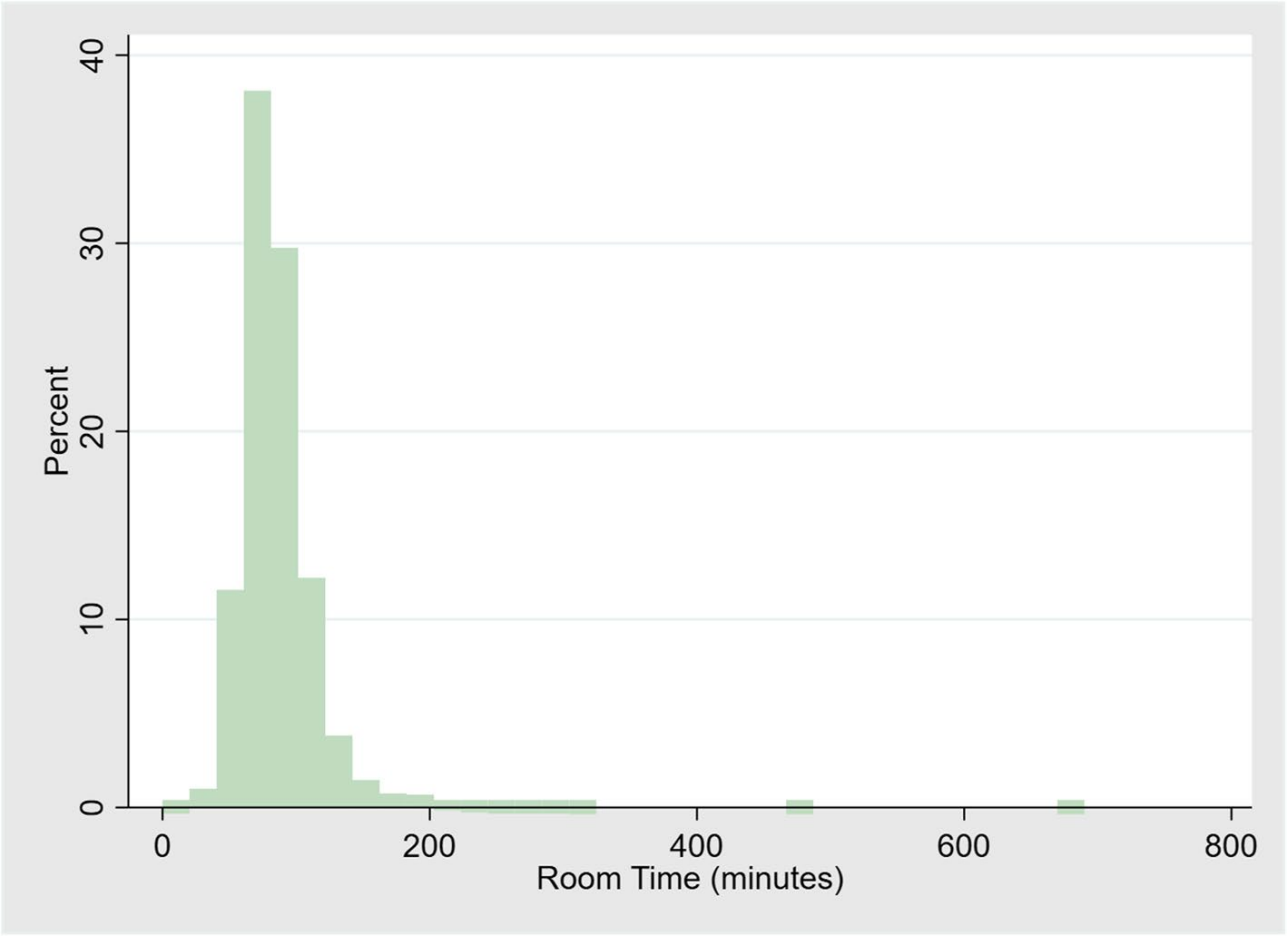
Poisson distribution of duration of operating room time in minutes

Nine thousand six hundred twenty-six deliveries occurred during the study period, 3,092 of which were CD (cesarean rate 32.1%). There were 4,017 total OR activities in the study period for 3,592 patients. 1,801 (50.1%) were unscheduled cases. Table 1 describes patient demographic information and outlines the types of procedures performed. Of these procedures, there were 3,092 CDs, of which 1,976 (63.9%) occurred between peak hours (07:00–19:00) (Fig. 2A). 63.8% of the time, two or more ORs were simultaneously running for up to 30 min (Fig. 2B). Total OR time followed a Poisson distribution (Fig. 3).

**Table 1:** Demographic information, procedure types, and frequency of each procedure. There were 4,017 total procedures for 3,592 patients in the study period

Attribute	Mean ± Standard Deviation		
Age (years)	30.9 ± 3.1		
	***n***		**Percent %**
ASA PS			
2	2751		76.6%
3	814		22.7%
4	27		0.8%
ASA PS “E” designation	1801		50.1%
Race			
White	2444		68.8%
Black	730		20.6%
Other	376		10.6%
Total	3550		
Obese (BMI ≥ 30 kg/m^2^)	2087		61.2%
Morbidly Obese (BMI ≥ 40 kg/m^2^)	553		16.1%
**Procedure**	***n***	**Percent %**	**Mean ± Standard Deviation** **Anesthesia Time (minutes)**
Cesarean Delivery	3092	77.0%	103.3 ± 35.5
Postpartum Tubal Ligation	513	12.8%	105.0 ± 36.1
Vaginal Delivery (twins, double set-up)	110	2.7%	77.8 ± 26.8
D&C, retained placenta	88	2.2%	73.1 ± 25.1
External Cephalic Version	79	2.0%	79.3 ± 27.3
Cerclage	25	0.6%	55.0 ± 18.9
PUBS/Laser/RFA/all fetal procedures	24	0.6%	81.5 ± 28.0
Emergency set-up with monitoring for non-reassuring fetal heart tones	21	0.5%	46.2 ± 15.9
Laceration Repairs	21	0.5%	87.1 ± 30.0
Cystoscopy	21	0.5%	209.3 ± 72.0
Abdominal hysterectomy	11	0.3%	281.3 ± 96.8
B-lynch	6	0.1%	145.2 ± 49.9
Laparotomy	6	0.1%	149.8 ± 51.5

*SD* standard deviation, *ASA* American Society of Anesthesiologists Physical Status, *E* emergency, *BMI* body mass index, *D&C* dilation and curettage, *PUBS* percutaneous umbilical surgery, *RFA* radiofrequency ablation, *B-lynch* Balogun-Lynch suture

Over 24-h, arrival rate λ was 0.34 patients per hour; service rate per server team (µ) was 0.87 patients per hour. Between peak hours 07:00 to 19:00, arrival rate λ increased to 0.45 patients per hour; service rate per server team (µ) remained unchanged at 0.87 patients per hour.

Table 2 shows results of the analysis where number of server teams (s) dedicated to OR activity was varied between 2 to 5 during peak hours. Utilization ρ varied from 10% (5 server teams) to 25% (2 server teams). No-wait probabilities Wq(0) increased to > 99% with more server teams in the system; staffing with 2 server teams resulted in Wq(0) = 91.2% during peak hours (07:00–19:00). Average wait time in the system (Ws) increased with a lower number of server teams. Over 24 h, the probability of no patients in the system was P_0_ = 0.61, while the probability of one patient in the system was P_1_ = 0.23, and the probability of 2 or more patients in the system was P_≥2_ = 0.05 (P_3_ = 0.006). However, between peak hours 07:00–19:00, when the number of server teams = 3, the probability of no patients in the system P_0_ = 0.48, while P_1_ = 0.25, and P_≥2_ = 0.07 (P_3_ = 0.01, P_4_ = 0.002, P_5_ = 0.0003) and utilization was ρ = 0.17. At server team numbers between 3 and 5, Wq(0) was within the stated goal of at or above 99%. With server number = 3 and number of ORs in use in the system (n) = 4 and 5, P_n_ was < 1%; similar findings were seen when server team number = 4. At a server number of 5, no further changes were noted in the probability that n patients require simultaneous OR service (P_n_) when n = 4, indicating that 5 dedicated server teams and more than 4 ORs did not further improve wait times or other queueing parameters (Table 2). Variable symbols and definitions are summarized in Table 3.

**Table 2:** Results of queueing analysis for obstetric operating room events during peak hours (07:00 to 19:00)

Queue station	Obstetric operating room							
λ	0.451							
µ	0.865							
Server Teams	2		3		4		5	
P_0_	0.479		0.479		0.479		0.479	
Ls	0.552		0.524		0.521		0.521	
Lq	0.031		0.003		0.000		0.000	
Ws	1.225		1.162		1.157		1.156	
Wq	0.069		0.006		0.001		0.000	
Wq(0)	0.912		0.986 ^**a**^		0.998 ^**a**^		1.000	
*ρ* (%)	0.261		0.174		0.130		0.104	
***Steady-state distribution and operating characteristics***								
***Server Teams***	***2***		***3***		***4***		***5***	
	***n***	***P***_***n***_	***n***	***P***_***n***_	***n***	***P***_***n***_	***n***	***P***_***n***_
	0	0.478766	0	0.478766	0	0.478766	0	0.478766
	1	0.249549	1	0.249549	1	0.249549	1	0.249549
	2	0.065037	2	0.065037	2	0.065037	2	0.065037
	3	0.016950	3	0.011300	3	0.011300	3	0.011300
	4	0.004417	4 ^**a**^	0.001963	4 ^**a**^	0.001472	4	0.001472
	5	0.001151	5 ^**a**^	0.000341	5 ^**a**^	0.000192	5	0.000153
	6	0.000300	6	0.000059	6	0.000025	6	0.000016
	7	0.000078	7	0.000010	7	0.000003	7	0.000002
	8	0.000020	8	0.000002	8	< 0.000001	8	< 0.000001
	9	0.000005	9	< 0.000001				
	10	0.000001						
	11	< 0.000001						

λ = arrival rate

µ = served rate

P_0_ = probability that the system is empty

Ls = average number of people in the system

Lq = average length of the queue or the average number of people in a line waiting for service

Ws = average time for a patient in the system (waiting time plus service time)

Wq = average time spent in queue

Wq(0) = probability that the time spent in the queue is 0

ρ = utilization factor for entire system

n = number of patients in the system / number of operating rooms in use in the system

P_n_ = probability that n patients require simultaneous operating room service

^*^Parameters at which Wq(0), n, and P_n_ are all optimized in that the probability of zero wait time is equal to or above 99% and probability that n patients requiring simultaneous operating room service is < 1%. At server team number 5, no further changes in P_n_ when n = 4 are noted, implying no added benefit to the system when server team number is 5

**Table 3:** Variable symbols and definitions

Variable symbol	Definition
λ	arrival rate
µ	served rate
P_0_	probability that the system is empty
Ls	average number of people in the system
Lq	average length of the queue or the average number of people in a line waiting for service
Ws	average time for a patient in the system (waiting time plus service time)
Wq	average time spent in queue
Wq(0)	probability that the time spent in the queue is 0
ρ	utilization factor for entire system
n	number of patients in the system / number of operating rooms in use in the system
P_n_	probability that n patients require simultaneous operating room service

## Discussion

The primary results of our study support that QTA can be used at specific hospital sites to gauge appropriate anesthesia operating room staffing for maternity care. Our study found that in our high-volume Level 4 Maternity Center with a 9,626 annual birth rate and 32% CD rate with standard wait time tolerance, 3 to 4 dedicated operating room server teams and 4 to 5 dedicated operating rooms should be available. These findings are consistent with our current clinical practice that factors staff availability and room turnover needs, with infrequent congestion periods. Applying QTA in this setting was feasible and provided insights into staffing efficiency, surge capacity, and preparedness. Our results may be helpful to other institutions of similar sizes and operational scope. Our methods of QTA can translate to other maternity centers of different sizes.

Studies have used QTA to describe staffing efficiency in maternity centers in general terms. In one study, a rough approximation of patient flow and predicted probability distribution for patients at nighttime was generated using inpatient census data [8]. This study shed some light on capacity planning but did not specifically address OR activities for maternity care. Another study [9] found that QTA could effectively predict patient flow in a maternity center and then performed analyses to observe the impact of CD rate reductions on hospital resource requirements, including the financial impact of these reductions. However, this study was not designed to speak specifically to OR and anesthesia staffing requirements in maternity centers. To our knowledge, ours is the first study to use QTA to identify specific staffing and space needs for birth center OR requirements. Our data and methodologies can be helpful to other institutions seeking to improve efficiency or anticipating changes in service needs.

Prior work in trauma hospitals and emergency departments – to which obstetrics operations are often compared – has used mathematical approaches to define manpower needs. In one trauma center study [10], mathematical modeling was used to define neurosurgical trauma coverage. After thousands of iterations for sample sizes of 25 to 300, a conclusion was made that mandatory neurosurgical backup for trauma centers performing fewer than 25 neurosurgery procedures per year was not necessary. We are compelled to note that regardless of the volume of procedures performed, a determination to provide coverage must be balanced and driven by patient safety and quality rather than workload efficiency alone. QTA is frequently used in the emergency department to predict delays, identify bottlenecks, utilization ratios, and wait times [11]. Each organization should create its own activation thresholds for staffing, including but not limited to in-house and out-of-house backup services, and ideally base these decisions on optimal safety parameters. In obstetrics, utilization (ρ) has not been defined. Our data shows that ρ changes depending upon the time of day with the introduction of elective cases on top of urgent/emergent cases that arise consistently throughout the day. Setting a ρ = 1 could place a system in “maximal” efficiency but would lead to intermittent backlogs. Backlogs may not be tolerable in obstetrics practices where timely access to an operating room means the difference between good and bad maternal or neonatal outcomes.

Our methods provide a rational framework and a case example by which maternity centers and anesthesiology leadership can make informed staffing decisions based on center workload. In areas where regionalization of maternity care may be occurring based on ACOG [2] recommendations, QTA can be used to identify anticipated resource needs based on volume data and baseline assumptions in wait time tolerances. This approach may be more informative than an approach that sets benchmarks by query of different institutions of similar or different sizes; the latter approach is limited in that each hospital has differences in operational characteristics, including state regulatory requirements for supervision or direction, or state laws that impact patient characteristics and volumes for anticipated operative procedures. We believe this approach is also helpful because it is driven by measurable institutional data rather than subjective assessments of right-sized staffing, and that the analyses can adapt over time with changes in service characteristics and volume. Notably, we set the ρ to above 0.10 to buffer demand fluctuations, but we should note that higher and lower target utilization rates may be appropriate based on factors like service level agreement with contracted providers like anesthesiology groups, cost constraints, and patient expectations.

One of the main purposes of using QTA is to identify any flaws in the current system and suggest how to address systems issues to achieve a balanced system that is both efficient and affordable. In the context of obstetric ORs, QTA can be used to identify scheduling flaws or inefficiencies, optimize resource allocation for nurses and other staff, reduce wait times for both scheduled and urgent cesarean deliveries or other time-sensitive activities, and ultimately improve the overall patient experience. QTA enables data-driven decision-making and continuous improvement in healthcare operations.

This study facilitates further research in optimal staffing and surge capacity response in both urban and non-urban hospitals. Additional models using labor and delivery suite data can address staffing issues for non-OR obstetric anesthesia clinical activities and efficiencies. In our clinical practice, in addition to 2 dedicated obstetric anesthesiologists, during peak hours we have 2 QHOP (i.e., residents, fellows, or nurse anesthetists) staffing the labor and delivery suites in addition to 3 QHOP allocated for OR cases, for a total of 5 QHOP always available during peak hours, not including the attending anesthesiologists. This number only decreases to 4 QHOP during non-peak hours. In our clinical experience, this resource allocation is sufficient to meet our obstetric patients’ needs in a timely fashion in both OR and non-OR activities. Our hospital currently enjoys high patient satisfaction ratings reported by Hospital Consumer Assessment of Healthcare Providers and Systems (HCAHPS) surveys, supporting our assertion that this staffing model effectively meets our needs. Further work is needed to identify how staffing and space parameters might change at other institutions that may have different volumes, arrival times, and wait time tolerances. Finally, individual hospitals may perform a sensitivity analysis and assign a dollar value to altering these probabilities.

There are limitations in our study. We only examined birth center OR activity and did not include labor and delivery room activity. Therefore, these findings do not apply to all birth center activities with which anesthesiology teams are involved, e.g., labor analgesia initiation and maintenance, hypotension treatments, complex care planning and discussions, teaching (for teaching institutions), and follow-up activities. A limitation of queueing models is the potential that waiting space may be limited, or the arrival rate is state-dependent – that is, people may be discouraged from entering the queue if a long line is observed. We believe these latter limitations are not present in our study because waiting space is not a limitation in admitting women to labor and delivery at our institution. Also, patients requiring urgent operative obstetric clinical care are not necessarily able to leave the queue. Our assumptions for 30 min decision-to-incision for unscheduled, non-emergent deliveries and 5 min for emergent deliveries, are based on general obstetric clinical performance to minimize risk for death or asphyxia in situations of prolonged fetal heart rate decelerations with or without preceding severe late or variable decelerations [12, 13]. We acknowledge the controversy that surrounds these time interval metrics [14–17]. Finally, our study is limited in that it is reflective of a high-volume, Level 4 maternity center, and thus our specific findings do not generalize to maternity centers with different queueing characteristics and service needs. However, we believe that our QTA methods are still valuable for those centers to identify optimal staff and space utilization. These methods are more useful than static workforce benchmark surveys because they accurately predict resource requirements based on each center’s specific patient flows.

In summary, QTA is a useful tool to benchmark birth center perioperative efficiency while upholding safety standards and factoring peaks and troughs of daily activity. QTA can be used for perioperative activity in maternity centers of all sizes. These data can inform individual hospital-level decisions in setting birth center OR staffing and space requirements, which are essential to maintain safe standards and efficient operations.

## Data Availability

Data supporting the findings of this study are available from the corresponding author, but restrictions apply to the availability of these data which were used under license for the current study so are not publicly available. The datasets used and analyzed during the current study available from the corresponding author on reasonable request.
